# Cross-sectoral genomic surveillance reveals a lack of insight in sources of human infections with Shiga toxin-producing *Escherichia coli*, the Netherlands, 2017 to 2023

**DOI:** 10.2807/1560-7917.ES.2024.29.49.2400264

**Published:** 2024-12-05

**Authors:** Ingrid HM Friesema, Menno van der Voort, Ben Wit, Angela HAM van Hoek, Maaike JC van den Beld, Coen van der Weijden, Eelco Franz

**Affiliations:** 1 Centre for Infectious Disease Control, National Institute for Public Health and the Environment (RIVM), Bilthoven, The Netherlands; 2 Wageningen Food Safety Research (WFSR), Wageningen, The Netherlands; 3 Netherlands Food and Consumer Products Safety Authority (NVWA), Utrecht, The Netherlands

**Keywords:** Shiga toxin-producing *Escherichia coli* (STEC), surveillance, One Health, zoonosis, food-borne disease

## Abstract

Shiga toxin-producing *Escherichia coli* (STEC) is a zoonotic pathogen associated with illness ranging from mild diarrhoea to haemolytic uremic syndrome (HUS) or even death. Cross-sectoral data sharing provides an opportunity to gain insight in reservoirs and sources of human infections and starting points for pro-active measures. Nevertheless, phylogenetic clustering of STEC strains from animals, food and human cases is low in the Dutch surveillance system. This is partly due to the substantial contribution of international travel and person-to-person spread in the STEC epidemiology. Furthermore, some STEC strains causing disease in humans may have a human reservoir. Although the main reservoirs and sources are included in the Dutch monitoring programmes, some animals and food products may be under-recognised as potential sources of human infections. More effort in investigating the role of other reservoirs beyond the well-known can provide a better understanding on STEC ecology in general, improving surveillance and source attribution, and ultimately provide better guidance for monitoring and source finding. This also implies having good diagnostics in place and isolates available for typing. Therefore, on the human side of the surveillance, the decision has been made to start isolating STEC at national level.

## Background

Shiga toxin-producing *Escherichia coli* (STEC) is a zoonotic pathogen associated with illness ranging from mild diarrhoea to haemolytic uremic syndrome (HUS) or even death [1,2]. As ruminants (especially cattle, goat and sheep) are the main reservoir, consumption of contaminated food of bovine, caprine and ovine origin, and contact with these animals or their faeces, are known transmission routes [3]. Nevertheless, the infection is often acquired abroad, and person-to-person transmission is also reported regularly [4,5]. Besides the potentially severe clinical symptoms, STEC is a public health concern given its recognised potential to cause food-borne outbreaks. Therefore, many countries apply farm animal and food monitoring programmes as well as surveillance of human cases in order to assess risks, monitor circulating strains and detect as well as investigate outbreaks.

With the introduction of whole genome sequencing (WGS), a tool with excellent discriminatory power and robustness became available to serve risk assessment, monitoring, surveillance and outbreak investigations [6,7]. Combining WGS data from animal, food and human cases in a One Health surveillance context could provide a head start for outbreak investigation. Indeed, this has been very effective in source identification of *Listeria monocytogenes* [7-11] and *Salmonella enterica* outbreaks [12-14]. Genomic relationships were also found between STEC isolates obtained from food samples and human cases, for example in two outbreaks in Belgium [15]. Nevertheless, the success rate of cross-sectoral WGS-based surveillance in matching human patients with animal or food isolates appears to be lower for STEC than for *L. monocytogenes* and *Salmonella*, at least in the Netherlands. This raises questions about potential under-recognised sources responsible for STEC infections. In this perspective we evaluate the cross-sectoral Dutch WGS database for its ability to match patients to sources and suggest potential improvements for the STEC surveillance, with emphasis on the use of WGS within a One Health concept.

## STEC surveillance in the Netherlands

In the Netherlands, laboratories and medical doctors have to notify laboratory-confirmed STEC cases to the regional public health service by law. The public health service subsequently reports cases to the National Institute for Public Health and the Environment (RIVM). Since July 2016, notification criteria have been restricted to acute STEC infections with at least diarrhoea, vomiting, blood in stool or HUS [16]. Furthermore, laboratories are requested to send, if available, an isolate from the confirmed STEC cases to the RIVM for further typing. The number of notified cases varied between 436 and 731 over the years 2017 to 2023. In 2017, an isolate for typing was available for 54% of the cases, but that proportion decreased to 26–37% in the period 2018 to 2023. The main reason for this decline is the progressive replacement of culturing practices with PCR in the Dutch medical laboratories.

The Netherlands Food and Consumer Product Safety Authority (NVWA) is responsible for the implementation of farm animal and food monitoring programmes, and Wageningen Food Safety Research (WFSR) analyses the samples taken. The monitoring programme is based on risk and therefore focused on the most relevant pathogens (mainly those for which legal criteria exist) at different levels of the production chain such as farms, slaughterhouses, industry, wholesale, retail, and imported products at border control points. Besides manure, samples are mainly food products with a focus on meat (fresh and prepared/ready-to-eat), but also herbs, shellfish and vegetables. The number of samples taken for each level of production (in 2023, in total 3,515 food samples were analysed for STEC) can be found in the yearly reports of the Multi-Annual National Control Plan [17,18].

The STEC WGS data are mutually shared in a near real-time manner between RIVM and WFSR, with the aim of increasing the speed and success rate of source finding in case of (active) clusters. Clusters, consisting of sequences of two or more STEC isolates, are defined based on single linkage hierarchical clustering. Since STEC is genomically more diverse than *Listeria* or *Salmonella*, where it is common to use three to five alleles as a threshold for primary surveillance, we used seven allelic differences in both the surveillance and for the current article. The shared database contained a total of 3,345 sequenced isolates, from January 2017 up to November 2023 (see Table 1). Of these, 1,873 were collected from human cases and 1,472 originated from non-human sources. The three most common serotypes among human cases were O157:H7 (25%), O26:H11 (12%) and O146 (O146:H21 and O146:H28; 9%) accounting for 45% (844/1,873) of the isolates. In comparison, O146 (O146:H21 and O146:H28; 10%), O113 (O113:H4 and O113:H21; 6%), and O55:H12 (5%) were the three most common serotypes found in the non-human isolates, representing 21% (303/1,472). In total, 140 serotypes were present in the database, with 81 occurring in both patients and non-human sources, while 37 serotypes only occurred in patients and 22 serotypes only in non-human sources. Most common was the presence of only *stx2*, both in human (37%; 697/1,873) and non-human (43%; 626/1,472) isolates, while *stx1* was seen in 26% (482/1,873; human) and 35% (510/1,472; non-human), and the combination of *stx1* and *stx2* in 29% (534/1,873; human) and 20% (297/1,472; non-human). The *stx2* gene variant *stx*_2f_ was mainly seen in isolates from patients (8%; 157/1,873) and hardly in food (0.3%; 4/1,472). The *stx* profile was unknown for three human (0.2%) and 35 non-human (2%) isolates. The attaching and effacing gene was detected in 64% (1,191/1,873) of the human isolates compared with 14% (197/1,472) of the non-human isolates.

**Table 1:** Number of isolates and genetic clusters by source of isolate (human/non-human), the Netherlands, January 2017–November 2023 (n =3,345 isolates)

Clusters consisting of	Clusters		Total isolates		Human isolates		Non-human isolates	
	n	%	n	%	n	%	n	%
Human isolates only	170	60	511	15	511	27	NA	
Non-human isolates only	100^a^	35	231	7	NA		231	16
Both human and non-human isolates	15	5	41	1	22	1	19	1
Not part of a cluster	NA		2,562	77	1,340	72	1,222	83
**Total**	**285**	**100**	**3,345**	**100**	**1,873**	**100**	**1,472**	**100**

NA: not applicable.

^a^ Of these, 68 clusters consisted of isolates from meat products, 20 from faeces/manure, four from a combination of meat products and faeces/manure, one from vegetables, one cluster consisted of an isolate from a meat product and one isolate from a vegetable; the information on source was lacking for six clusters.

Only 15 WGS clusters (15/285; 5%) comprising both human and non-human isolates were identified (Table 1). Nine of these clusters consisted of one human and one non-human isolate, while the maximum size of a mixed cluster was five isolates (Table 2). The non-human isolates in these mixed clusters originated from beef products, manure of calves or sheep, carcasses of calves, and lamb. In most clusters, more than 6 months had passed between the sampling dates of non-human vs human isolates. The detection of these clusters did not lead to the start of an investigation because of the small size of the clusters and the long period between the sampling dates.

**Table 2:** Characteristics of the mixed clusters, the Netherlands, January 2017–November 2023 (n = 15 clusters)

Serotype	*stx*/*eae* gene presence	Cluster size	Human:non-human	Non-human source type	Year	Time between human and non-human isolates
O103:H2	*stx1*^a^; *eae*	5	4:1	Manure veal calves at farm	2018, 2019, 2022	5 months (n = 1) and 30–38 months (n = 3)
O117:H4	*stx2* ^a^	4	1:3	Carcass calves at slaughterhouse	2018–2021	20–39 months^b^
O157:H7	*stx1*+*stx2*; *eae*	4	3:1	Veal at retail	2017–2019	4 months (n = 2) and 15 months
O157:H7	*stx1*+*stx2*; *eae*	4	1:3	Manure cattle at farm and carcass calves at slaughterhouse	2017–2018	4–13 months^b^
O146:H21	*stx1*+*stx2*	3	2:1	Lamb at retail	2019, 2021	10–12 months^b^
O182:H25	*stx1; eae*	3	2:1	Manure veal calves at farm	2019, 2022	41–43 months
O8:H9	*stx2*	2	1:1	Beef minced meat at retail	2017, 2021	59 months
O26:H11	*stx2; eae*	2	1:1	Lamb at retail	2018–2019	14 months^b^
O98:H21	*stx1; eae*	2	1:1	Filet americain at retail	2017	< 1 month^b^
O103:H2	*stx1; eae*	2	1:1	Manure sheep at farm	2022–2023	15 months
O103:H2	*stx1; eae*	2	1:1	Carcass calves at slaughterhouse	2019–2020	17 months^b^
O111:H8	*stx1; eae*	2	1:1	Carcass calves at slaughterhouse	2021–2022	9 months^b^
O113:H21	*stx2*	2	1:1	Filet americain at retail	2018	< 1 month^b^
O117:H4	*stx2*	2	1:1	Filet americain at retail	2020	2 months^b^
O146:H21	*stx1+stx2*	2	1:1	Manure sheep at farm	2022–2023	12 months

^a ^One non-human isolate harboured *stx1* and *stx2*.

^b ^Sampling date(s) of the non-human isolate(s) precede sampling date(s) of the human case(s).

## Gaps in cross-sectoral STEC surveillance

Several potential reasons underlying the limited overlap between patient and non-human (animal and food) isolates can be postulated. Below we provide an overview of mutually not exclusive reasons and their different their contribution to the phenomenon.

### Travel-related and secondary transmission

A substantial part of the human infections are acquired abroad. With the exception of the years of the COVID-19 pandemic, 2020 and 2021, around a quarter of the notified STEC infections in the Netherlands are related to international travel, and thus will not be related to Dutch animals or food obtained in the Netherlands. It is therefore important that information on travelling in the week before illness onset is reported. 

Around 15% of the cases mention other persons with illness in their surroundings, although in most of those cases, this could not be confirmed microbiologically because the other persons were not tested, and thus not notified, or because isolates for comparison were not available. A meta-analysis indicated person-to-person spread as the second most important STEC transmission pathway after consumption of raw or undercooked meat [19], and a recent case–control study in the United Kingdom showed childcare occupations as a risk factor of infection for all STEC serotypes [20]. In addition, there are several reports about sexual transmission of STEC [21,22].

### Possible non-zoonotic STEC

It is increasingly recognised that Stx-producing human-adapted *E. coli* hybrid pathotypes (incl. enteroaggregative, extraintestinal, enteropathogenic and enterotoxigenic pathogenic *E. coli*) circulate and cause disease in humans and have hardly been detected in animals [23]. A notable example is the Stx-producing enteroaggregative O104:H4 strain causing an outbreak that started in Germany in 2011, which to date can sporadically be detected and very likely has a human origin [24]. Similarly, extensive comparative genomics of *stx*_2f_-carrying STEC from human infections, which largely were Stx-producing typical enteropathogenic *E. coli* (tEPEC), revealed that these isolates are most likely to have a human reservoir [25].

### Under-recognised animal reservoirs and food transmission pathways

Although ruminants, particularly cattle, are regarded as the main reservoir for STEC, there is evidence that dogs, fish, horses and pigs and some (wild) bird species including poultry are relevant spill-over hosts [3,4,26]. These animals are susceptible to colonisation by STEC, but in contrast to reservoir animals, they do not maintain the bacteria in the absence of continuous exposure. Implicitly, this means that there may be other epidemiologically relevant sources of human STEC infection beyond ruminants which are seldomly monitored or sampled. Vegetables (including sprouts) and fruit have also been related to STEC infections and large outbreaks [27-29]. Until now, outbreaks due to fresh produce have hardly been seen in the Netherlands. Nevertheless, contamination of fresh produce via nearby livestock farms is a realistic risk and should be kept in mind. Two other potential transmission pathways that have come to attention more recently are flour [30-33] and raw pet food [34-36]. Although the main reservoirs and sources are included in the Dutch monitoring programmes, adding fish, flour, poultry and raw pet food could enrich our surveillance system.

### Monitoring and sampling schemes

Potentially, there is a substantial impact of the design of monitoring and sampling schemes on the final collection of animal and food isolates that are compared with those of human infections. A lack of understanding of the diversity of relevant STEC sources (see previous paragraph) limits accurate risk-based sampling. In addition, the nature of STEC being a genomically extremely diverse pathogen reduces the likelihood of detecting identical patient and source strains. This applies especially in combination with relative long farm-to-fork chains of bovine meat products, where ample opportunities exist for diversification of the STEC population present at different points in the production chain. An examination whether the focus in the current monitoring programmes has an influence on whether STEC types are detected, and which ones, could lead to more insight. Also investigating the diversity of STEC types that can be found on farms, in individual animals and food would provide useful insight.

### STEC detection and isolation procedures

It is well known that STEC detection and isolation is cumbersome and does not always succeed in obtaining isolates for typing. In human diagnostics, the increasing trend towards molecular diagnostics without (attempts at) isolation creates the problem of receiving fewer STEC for typing, which subsequently results in loss of effective cluster and outbreak detection. To overcome this drawback, RIVM will start performing STEC isolation from patient faeces that tested positive in molecular diagnostics at medical laboratories, after which isolates will be further typed using WGS techniques. Another potential confounding effect might be the occurrence of mixed contamination of sources, complicated by potential horizontal exchange of virulence genes with commensal *E. coli* in the gut, because in general, only one isolate is retrieved and typed while in reality, multiple STEC types could be present.

## Conclusion

The absence of overlap of STEC strains from human and non-human sources in the Netherlands can have multiple reasons and causes. However, quantifying the impact of each possible reason and taking measures to reduce the impact is difficult. The most obvious factors are the substantial contribution of international travel and person-person spread in the STEC epidemiology. More efforts can be directed to comparing national sequences to international databases in order to infer the magnitude of relations to other geographical regions in relation to travel and/or imported food. More attention should be paid to identifying hybrid STEC strains and their epidemiology. In addition, some animals and food products may be under-recognised as potential sources of human infections. More effort in investigating the role of other sources beyond the well-known can provide a better understanding on STEC ecology in general, improving surveillance and source attribution, and ultimately provide better guidance for monitoring and source finding. All this also implies that sufficient attention must be paid to having good diagnostics in place and isolates available for typing.
